# Longitudinal Relationship Between Living Alone and Activity Engagement of Chinese Older Adults

**DOI:** 10.1093/geroni/igab046.3314

**Published:** 2021-12-17

**Authors:** Fengyan Tang, Ke Li

**Affiliations:** 1 University of Pittsburgh, Pittsburgh, Pennsylvania, United States; 2 University of Pittsburgh, Sandy Springs, Georgia, United States

## Abstract

The living arrangement of older adults plays a key role in their health status and psychological well-being. Yet the relationship between living arrangement and activity engagement remains unclear. Using data from three waves of the nationally representative China Health and Retirement Longitudinal Study (CHARLS) with a study sample of 7,479 respondents aged 60 or older, this study examined the effect of living alone on the frequency of activity engagement among Chinese older adults. Two types of activity engagement were examined in this study, including social interaction with friends and leisure activity (i.e., play chess, go to a sport or club). The multinomial logistic regression analyses were performed using generalized structural equation modeling (GSEM). Compared with those living with others, older adults living alone were older, more likely to be female and living in urban areas, and with fewer years of education and more functional limitations. The results also indicated that after controlling for a set of covariates, living alone status was significantly associated with the increased likelihood of high and medium frequency of both social interaction and leisure activity in reference to no engagement. This study not only improves the understanding of activity engagement preference of Chinese older adults who are living alone but also indicates the importance of improving community facilities and outdoor spaces to promote activity engagement among older adults.

